# Medicare modernization and diffusion of endoscopy in FFS medicare

**DOI:** 10.1186/s13561-017-0147-5

**Published:** 2017-03-09

**Authors:** Lee R. Mobley, Pedro Amaral, Tzy-Mey Kuo, Mei Zhou, Srimoyee Bose

**Affiliations:** 1 Georgia State University, 1 Park Place, Suite 700, Atlanta, GA 30304 USA; 2 Cedeplar - Universidade Federal de Minas Gerais, Belo Horizonte, Brazil; 3 University of North Carolina, Chapel Hill, USA

## Abstract

**Objective:**

To examine how FFS Medicare utilization of endoscopy procedures for colorectal cancer (CRC) screening changed after implementation of the Medicare Prescription Drug, Improvement, and Modernization Act (MMA) in 2006, which provided subsidized drug coverage and expanded the geographic availability of Medicare managed care plans across the US.

Data Sources/Study Setting. Using secondary data from 100% FFS Medicare enrollees, we analyzed endoscopy utilization during two intervals, 2001-2005 and 2006-2009.

**Study design:**

We examined change in predictors of county-level endoscopy utilization rates based on a conceptual model of market supply and demand with spillovers from managed care practices. The equations for each period were estimated jointly in a spatial lag regression model that properly accounts for both place and time effects, allowing robust assessment of changes over time.

**Data collection/Extraction methods:**

All Medicare FFS enrollees with both Parts A and B coverage who were age 65+, remained alive and living in the same state over the interval were included in the analyses. The later interval used a new cohort defined the same as the earlier interval. 100% Medicare denominator files were also used, providing county of address to use for county-level aggregation. The outcome variable was defined as county-level proportion of enrollees who ever used endoscopy over the interval.

**Principal findings:**

Endoscopy utilization by FFS Medicare increased, and became more accessible across the US. Medicare managed care plan spillovers onto FFS Medicare endoscopy utilization changed over time from a significant negative (restraining) effect in the early period to no significant effect by the later period.

**Conclusions:**

The MMA eased budget constraints for seniors, making endoscopic CRC screening more affordable. The MMA policies also strengthened managed care business prospects, and enrollments in Medicare managed care escalated. The change in managed care spillover effects reflects the gradual acceptance of endoscopic CRC screening procedures, as they emerged as the gold standard during the period.

## Background

Utilization of endoscopic procedures (colonoscopy, sigmoidoscopy) for colorectal cancer (CRC) screening is effective in preventing precancerous tumors from developing into cancer, however the utilization rate is lower than recommended guidelines [1–3]. In 2001, the Centers for Medicare and Medicaid Services (CMS) expanded Medicare coverage to cover colonoscopy for persons with average risk for CRC, in the face of an emerging body of cost-effectiveness research on endoscopy screening for CRC and evidence recommending use of colonoscopy [4]. Over the next six years, utilization of colonoscopy diffused rapidly across FFS Medicare markets, and came to dominate the endoscopy services. At first, the expansion occurred in those markets with more favorable business prospects, and was slower to diffuse to minority-dominated areas [5]. Although the expansion in coverage for endoscopy helped improve uptake of endoscopic screening procedures, it still left the beneficiary facing substantial out-of-pocket copayments and facility fees, and utilization rates were suboptimal according to emerging screening guidelines.

In 2006, the implementation of the Medicare Prescription Drug, Improvement, and Modernization Act (MMA) offered subsidized prescription drug packages to seniors, available to both traditional fee-for-service (FFS) enrollees and managed care plan enrollees. The savings afforded by the drug plan coverage might have loosened budget constraints for many seniors, perhaps making the endoscopy copayments more affordable. Thus it is reasonable to expect that there might have been an increase in demand for endoscopic CRC screening after implementation of the MMA in 2006.

**Fig. 1:**
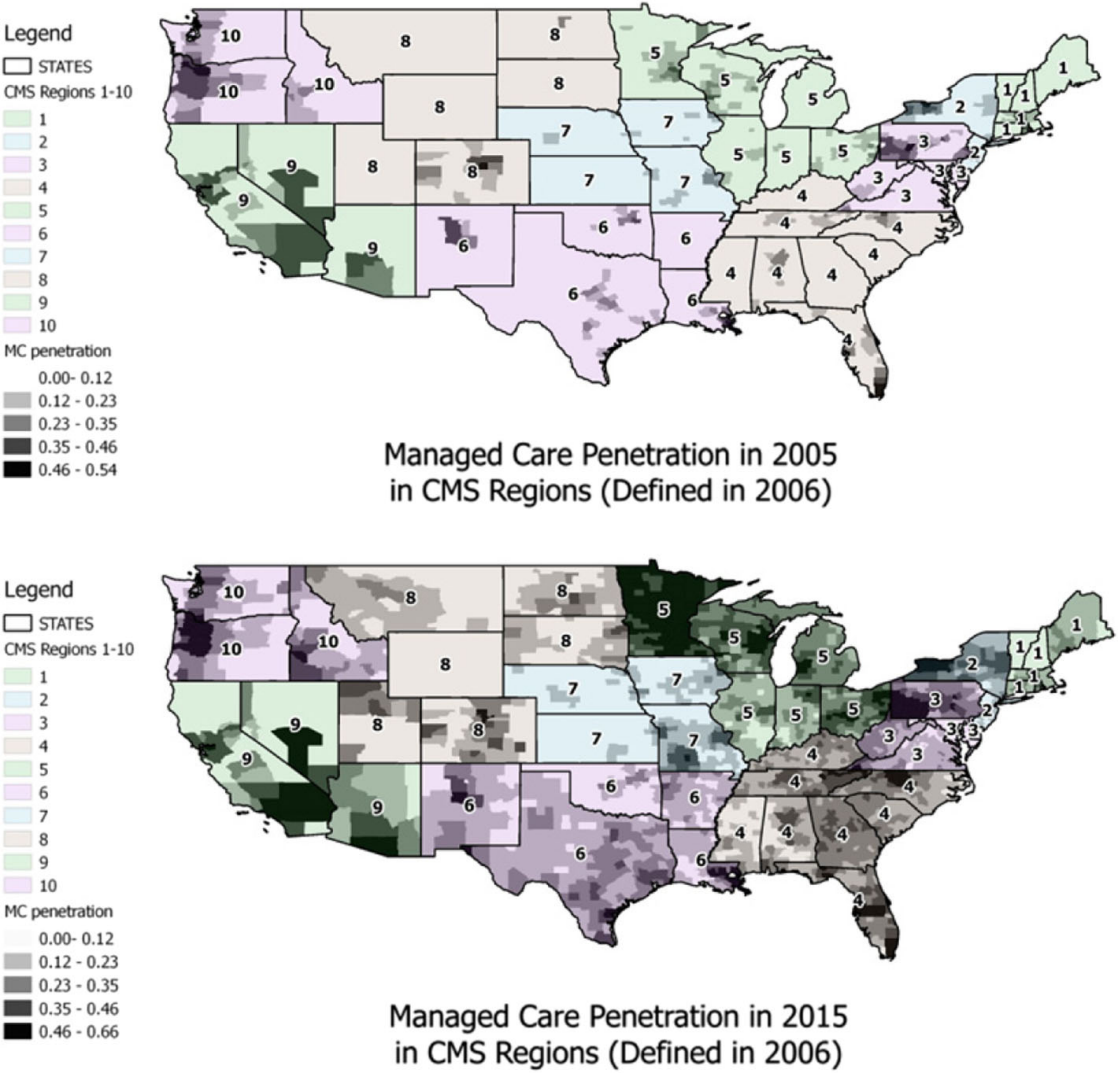
Medicare Managed Care Penetration Across the Ten CMS Regions, Before and After the MMA

In addition, the MMA led to considerable expansion in the Medicare managed care program across the US. The Act renamed the Medicare + Choice program the Medicare Advantage (MA) program, and made it much more attractive to seniors by adding prescription drug coverage to all MA plans [6]. CMS also re-defined the so-called ‘CMS Regions’ into ten new areas configured to enhance expansion of MA plans into all areas of the US ([7]; CMS [8]). The Medicare managed care penetration rate increased from 15% in 2000 to 24% in 2010 and continued to increase thereafter [9]. Figure 1 shows the penetration by Medicare MA plans in 2005 (before The Act was implemented), and in 2015 (most recent data available) across the ten CMS regions.

Managed care plans have been reputed to disseminate best-practice guidelines and encourage use of preventative services with established cost-effectiveness evidence. Managed care penetration may have helped encourage the diffusion of colonoscopy as a CRC screening procedure. In 2008, colonoscopy was recommended as one of the primary colorectal cancer screening tests by the United States Preventive Services Task Force, following a systematic review of studies demonstrating colonoscopy as a cost-effective CRC screening procedure [10–12]. Since then one might expect that managed care practices would embrace and disseminate this information, resulting in an increase of colonoscopy utilization for CRC screening. Such a phenomenon may also spill over to non-managed care enrollees, as prior studies have shown managed care practices influence and spill over onto other market constituents, including Medicare FFS enrollees [13, 14]. For example, using data from 1999 and 2001-2006, two studies found modest spillover effects from MA penetration on FFS Medicare utilization of endoscopy for CRC screening [5, 15]. Therefore, it is reasonable to expect that the demand for endoscopic CRC screening increased for FFS Medicare enrollees through spillover effects from MA penetration after implementation of the MMA.

In this study, we exploited the natural experiment provided by the MMA implementation in 2006, to determine how the changes in market conditions during this decade predicted utilization of endoscopic CRC screening among the aged. The focus is on the traditional FFS Medicare enrollees for whom complete medical claims exist for study. Relaxation of personal budget constraints due to newly available subsidies, and market spillover effects emanating from managed care expansion are expected to increase both supply and demand for these services.

## Methods

### Conceptual model

We adapt the conceptual model used in published studies which included a comprehensive set of supply and demand factors that would determine the feasibility of establishing endoscopic CRC screening services in a given market [5, 16]. Acquisition of endoscopy equipment and the necessary training to use it will depend on the expected return on investment, which is a function of market conditions. Observed utilization rates will depend on various market factors, summarized in Table 1, which describes the variables we use to capture these aspects.

**Table 1:** Market conditions fostering FFS Medicare utilization of endoscopy services, and variables used in modeling

	2001-2005		2006-2009		
	Mean	St. Dev	Mean	St. Dev	Source
Dependent Variable: Avg. Annual % Endoscopy Utilization	7.4	1.2	7.9	1.3	100% of traditional FFS Medicare endoscopy claims
Market Demographics of the Medicare population (%)					
Age < 65	16.9	6.1	18.4	6.1	100% Medicare denominator files, averages 2001-2005; 2006-2010
Age 65-74	44.5	3.7	43.5	3.9	
Age 75-84	28.6	3.6	27.2	3.5	
Age 85+	9.9	2.4	10.9	2.8	
Female	55.0	2.8	54.2	2.5	
Caucasian	90.6	13.2	90.2	13.5	
African American	7.3	12.7	7.5	12.9	
Hispanic	1.0	3.2	1.0	2.9	
Asian	0.3	0.8	0.3	1.0	
American Indian and other races/ethnicities	0.8	4.3	1.0	4.9	
Dual/ESRD/disabled benefits	20.9	10.7	27.9	10.6	
Acculturation and educational attainment of the market population, and area poverty:					
Persons Aged 65+ with little or no English language ability (2000)	14.3	14.7	14.3	14.7	US Census 2000; American Community Survey, 2006-2010; Census SAIPE
Persons aged 65+ with graduate or professional degrees (2000; 2006-2010)	4.4	2.8	5.7	4.0	
Average poverty in the area over the past 20 years (1990-2000; 1995-2005)	14.8	6.7	14.1	5.7	
Market size, which affects pace of return on investment:					
Population density (2000, 2005) per sq. mile	235.1	1661.8	242.8	1713.4	Area Health Resource Files
Percent of the population with Medicare Part A benefits (2001, 2006)	14.1	2.0	14.8	2.0	
Health market conditions:					
Prevalence of managed care plans: Medicare managed care penetration, lagged 1 year (2000, 2005)	4.9	9.7	4.6	8.4	RTI Spatial Impact Factor Database
Prevalence of endoscopy providers: average distance from FFS claimants to closest provider (2001, 2006)	10.4	8.8	11.7	9.2	
Competition among endoscopy providers (2001, 2006) index, where 1 = perfect and 0 = no competition	0.020	0.046	0.021	0.052	

From the perspective of determinants of market potential, we use demographic factors defined for all Medicare eligibles (including those under age 65) from the Medicare denominator file to characterize the age distribution, race or ethnicity, and vulnerability in the Medicare population. Vulnerability is defined as eligibility for additional benefits to assist the low income (who may be dually eligible for Medicare and Medicaid benefits), the disabled, and those with end-stage renal disease (ESRD). This measure of vulnerability also captures some degree of medical need or comorbidity. Areas with higher percentages of vulnerable beneficiaries are not expected to be as manageable or as profitable for managed care plans that were reluctant to expand into all markets after experimental demonstrations by CMS offered substantial subsidies [17]. The acculturation, educational attainment, and area poverty variables capture aspects of demand for the services. The first two are defined for persons aged 65+, while area poverty is defined as average area poverty over two decades. Better educated elderly with good English language skills are expected to have a better grasp of the benefits and a greater demand for endoscopic screening. Persistently poorer areas are not expected to be attractive to entry by endoscopy providers. Market size, which affects the pace of return on investment, is reflected in population density and the percent of the population with Medicare Part A benefits. We surmise that the higher the percentage of the population with Medicare Part A benefits, the more important is Medicare as a demand segment in the market. Health market conditions are reflected by the geographic density of endoscopy providers, measured as the average distance among FFS claimants to providers closest to their ZIP code of residence, and a competition index among endoscopy providers (an inverse Herfindahl index, where 0 = no competition and 1 = maximal competition). Another health market condition factor is the prevalence of Medicare managed care plans, which may have spillover effects on FFS enrollee utilization rates.

### Managed care spillovers

There has been sustained interest over the years in the impacts of managed care plans on other market participants, the so-called ‘spillover effects’ of managed care. Defined as changes in financial incentives, physician practice patterns, costs, or the diffusion of new technology relative to what might occur in markets with little managed care influence - spillovers have been examined empirically for over twenty years [5, 13, 14, 18–24].

Changes in practice patterns for a substantial proportion of insured patients can spill over to people who are not insured by the managed care plans (including the FFS Medicare population) who are seen by the same physicians influenced by the managed care practices and protocols. Also, individuals not enrolled in managed care plans might compare prescribed treatment options with their peers who are enrolled in managed care plans. These behavioral spillovers among people and physicians ensure that managed care plans can impact the way medicine is practiced in their markets. With particular relevance to this study, managed care spillovers can impact adherence to CRC screening guidelines by patients, irrespective of whether they are enrolled in managed care plans.

To obtain robust and reliable estimates for these spillovers onto FFS Medicare utilization, empirical models must deal with sources of selection bias that could influence the estimates [5, 13]. Selection effects can be related to the relative generosity of MA plan versus FFS plan payment rates, the fact that wealthier elderly would have to give up supplemental coverage to enroll in managed care, the benefits to lower income elderly of obtaining ‘free’ Part B coverage by enrolling in managed care plans, and other socio-economic factors varying from place to place [5, 18, 25–27]. These socio-economic factors are expected to impact the demography of enrollment into MA plans, and the specifics of particular markets that MA plans may choose to enter. Factors among the Medicare population such as lower income or greater morbidity, age distribution, and racial or ethnic concentrations that vary from place to place are all factors that may have impacted where MA plans originally entered and attracted enrollees. After the MMA implementation in 2006, MA plans spread across the US to enter new markets not previously served, where spillover effects might reach fertile new ground. So there are many reasons to expect changes over time in the ecological model of county-level utilization of endoscopic CRC screening by the FFS Medicare population.

### Analysis samples, outcomes, and contextual factors

To examine how the MMA may have influenced endoscopic CRC screening utilization for the Medicare FFS enrollees, we constructed the population-based study sample in two cohorts based on the 100% FFS insured Medicare enrollees. First, we included the entire Medicare FFS population aged 65+ each year during 2001-2005, or 2006–2009. We then excluded all persons who did not have *traditional FFS Medicare coverage* (defined as both Parts A and B coverage for at least 11 months of the year) for all years in each interval. We excluded people who died or who moved to a different state during the interval, and a new cohort was derived using these criteria for the second interval. Current CRC screening guidelines for persons of average risk from the USPSTF recommend fecal occult blood testing (FOBT) every year, sigmoidoscopy every 5 years, and colonoscopy every 10 years. We focus only on the diffusion of endoscopy services because they are costly to provide and their availability is expected to fluctuate with market conditions. Following the approach in a previous study, we focus on ‘any utilization’, rather than attempting to ascertain optimal utilization patterns for each beneficiary, which is beyond the scope of this paper [5]. Focusing on ‘any utilization’ allows us to ascertain availability and diffusion of the services as predicted by market influences.

We first created an indicator for whether or not an individual had *ever used either of the services* over the interval. Services were defined using a comprehensive list of medical procedure codes consistent with other studies of endoscopic technology utilization for CRC screening (G0104,G0105,G0121,44388-44397,45300, 45305, 45308, 45309, 45315, 45317, 45320, 45327,45330,45331, 45333-45335, 45338-45342, 45345, 45355, 45378-45387, 45391, 45392) [2, 5, 15]. Procedures include, among other things, ‘screening with polyp removal and biopsy’, which insurers view as ‘diagnostic’. We include these because this more complex endoscopic procedure is prescribed by the endoscopy guidelines to meet the gold standard of screening for CRC. If the person used endoscopy more than once, the county of residence at first use was kept as the county of record for the analysis.

The sum of these person-level endoscopy utilization indicators by county is the numerator used in creating the county proportion of all traditional FFS Medicare enrollees (defined above) who had ever used one of the services over the interval. Because the early period is 1 year longer than the late period, and we desire a fair comparison of changes in utilization rates over time, we created the average annual utilization rate in each period by dividing the multi-year construct by the number of years it spanned. Thus the outcome variable of interest is the average annual utilization rate in the county for endoscopy services used during 2001-2005 or 2006-2009, among traditional FFS Medicare enrollees. The county-level proportion was defined separately for each county and time interval, and converted to a percentage for use in the analysis.

For the market contextual factors, we used 100% Medicare population demographic information from the Medicare denominator files to describe characteristics of beneficiaries, including age groups, sex, race or ethnicity, and vulnerability (Dual, disabled, ESRD) status. Averages over the intervals were used to represent these compositional factors. Other factors describing local market conditions were drawn from the U.S. Census and American Community Survey, the Area Health Resources Files, and the RTI Spatial Impact Factor database (see Table 1). These ecological variables together reflect aggregate market conditions in all counties across the continental United States, over the two time periods. Managed care penetration was defined for a one-year lag prior to each interval (2000 for 2001-2005; 2005 for 2006-2009) to reduce potential endogeneity. This variable was constructed from the CMS Geographic Service Area files, which provide the county number and proportion of enrollees in various types of plans (including managed care plans) by county.

### Descriptive analyses

**Fig. 2:**
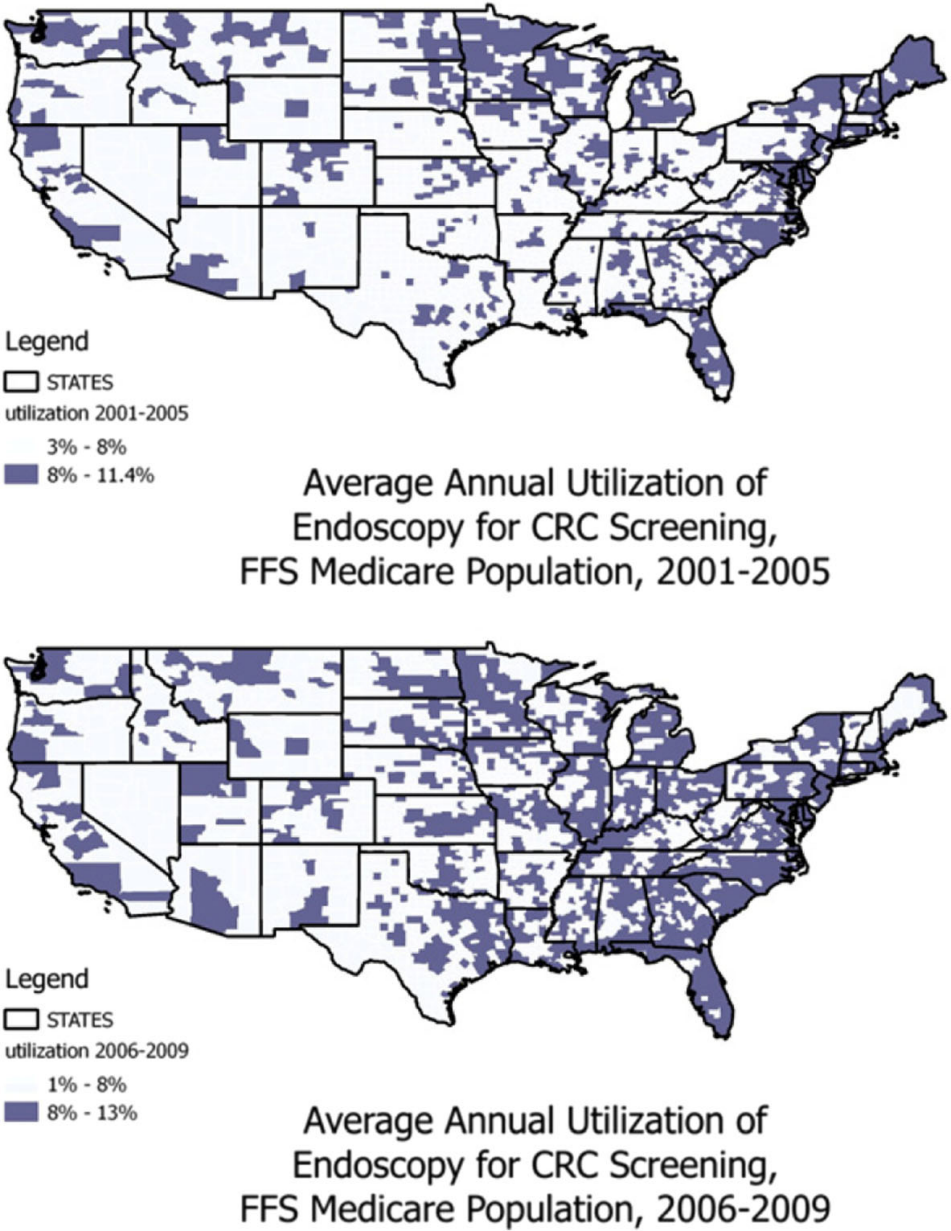
Average Annual Utilization of Endoscopic CRC Screening in FFS Medicare, in Two Periods Pre- and Post MMA of 2006

We calculated descriptive statistics for the variables we included in the statistical models, for the early (2001-2005) and late (2006-2009) periods, summarized in Table 1. In addition, we used mapping of the average annual utilization rates by county in each period to discern whether there was apparent diffusion of these services over the geography of the US (Fig. 2). Using the same cutpoints in the two figures allows a fair comparison over time of the average annual percent of FFS Medicare enrollees ever using these services each period. It is evident from a comparison of the two periods that average utilization increased over time and diffused or spread out over more geographic areas over time, as endoscopy became an accepted component of the gold standard for CRC screening.

### Statistical analysis

To estimate the associations between ecological factors and market outcomes, and how these may have changed over time, the challenge is to simultaneously handle two data correlation issues: (1) adjacent county observations on the screening utilization outcome may be spatially correlated, and (2) the county observations themselves may be correlated over time. Both situations may lead to reduced efficiency and reliability of statistical inference, and the first may result in spatial multiplier bias. We use the spatial seemingly unrelated regression (SSUR) empirical model that deals with both of these aspects.

First, we expected to see evidence of statistically significant spatial autocorrelation. Spatial autocorrelation across adjacent areas in outcomes related to public goods or preventive health care services that are expensive to provide is well documented in the empirical literature [28–32]. Because the decision regarding whether to establish endoscopy services in a county may be affected by prevalence of these services in adjacent counties, we expect there will be spatially correlated errors in the ecological models. Ignoring this is equivalent to falsely assuming that observations (county utilization rates) are statistically independent, which is a standard assumption under ordinary least squares (OLS) regression. This can lead to either efficiency bias, parameter bias, or both [33]. Recent papers have shown that ignoring spatial spillovers can yield highly inflated estimates of the marginal impact of living in rural poverty on preventive care service utilization by the elderly, misleading antitrust prescriptions, and inflated estimates of managed care spillover effects [5, 31, 32]. These misspecification effects can be corrected using a spatial regression model.

The second problem facing the ecological model is the possibility that area rates may be correlated over time. Ignoring this source of similarity or redundancy in the data inflates statistical significance. To reliably ascertain whether observed changes in parameter estimates over time are statistically significant, it is important to use an estimation setup that pools the two time periods and allows the two cross-sectional equations to have correlated errors over time. Thus, in estimation, we need an empirical approach that can deal with both spatial and time correlation. We adopt the seemingly-unrelated regressions (SUR) approach pioneered by Zellner [34], and expanded to include spatial autocorrelation by Anselin [33]. We used the residuals from OLS regression as diagnostics to test for spatial correlation and determine the best spatial model to employ. We then estimated a spatial lag model specification to perform the spatial regressions over the early (2001-2005) and late (2006-2009) time periods, incorporated within a seemingly unrelated regression (SUR) framework, which allowed us to pool the two equations over time [33, 35, 36]. All models were estimated using PySAL, a Python library for spatial analysis developed by the GeoDa Center for Geospatial Analysis and Computation [37]. The programming code for the spatial SUR models is now publicly available for general applications as part of PySAL, as described in a recent applied spatial econometrics textbook [36].

The spatial SUR (SSUR) model allows for spatially correlated error terms within each equation and across equations, with separate parameter estimates for each time period. To assess diffusion effects, we performed parameter-specific Wald tests to test for the stability of parameters across time. The null hypothesis under the Wald test is that parameters are stable (do not change significantly) over time. When this hypothesis is rejected, we can conclude that a significant change over time occurred; these are indicated with asterisks on the variables named in Table 2. The PySAL software also provides a Lagrange Multiplier test to assess whether the SUR simultaneous-equation estimation significantly improves the efficiency of the effect estimates (relative to an unpooled model).

**Table 2:** Estimation results, using ordinary least squares and spatial seemingly unrelated regression models

	OLS Model, 2001-2005		OLS Model, 2006-2009		SSUR Model, 2001-2005		SSUR Model, 2006-2009	
	Coef.	*P* > |t|	Coef.	*P* > |t|	Coef.	*P* > |t|	Coef.	*P* > |t|
Age 65-74^a^	0.013	0.112	-0.019	0.123	0.012	0.077	-0.016	0.144
Age 75-84^a^	**0.059**	**0.000**	**-0.037**	**0.011**	**0.035**	**0.000**	**-0.042**	**0.002**
Age 85 + ^a^	**-0.110**	**0.000**	**-0.182**	**0.000**	**-0.077**	**0.000**	**-0.185**	**0.000**
Female^a^	-0.002	0.850	**0.120**	**0.000**	-0.002	0.825	**0.121**	**0.000**
African American	**0.019**	**0.000**	**0.018**	**0.000**	**0.016**	**0.000**	**0.016**	**0.000**
Asian	-0.042	0.127	0.005	0.851	-0.022	0.358	0.020	0.430
Hispanic^a^	**-0.037**	**0.000**	-0.001	0.960	**-0.025**	**0.000**	0.002	0.877
all other^a^	**-0.016**	**0.001**	**-0.023**	**0.000**	**-0.012**	**0.004**	**-0.023**	**0.000**
Population density	0.000	0.989	0.000	0.481	0.000	0.979	0.000	0.539
Poor English	-0.002	0.079	**-0.004**	**0.028**	-0.002	0.087	**-0.003**	**0.031**
Graduate^a^	**0.087**	**0.000**	**0.035**	**0.000**	**0.065**	**0.000**	**0.023**	**0.000**
Importance Medicare^a^	**0.030**	**0.003**	**0.046**	**0.000**	**0.022**	**0.014**	**0.044**	**0.000**
Average Poverty	**-0.032**	**0.000**	**-0.026**	**0.001**	**-0.030**	**0.000**	**-0.019**	**0.009**
Distance endoscopy	**-0.026**	**0.000**	**-0.021**	**0.000**	**-0.021**	**0.000**	**-0.017**	**0.000**
Dual/ESRD/disabled^a^	**-0.024**	**0.000**	**-0.041**	**0.000**	**-0.021**	**0.000**	**-0.043**	**0.000**
MA plan penetration	**-0.005**	**0.009**	-0.004	0.182	**-0.007**	**0.000**	-0.004	0.073
Competition endoscopy	**1.325**	**0.007**	**1.073**	**0.041**	**1.342**	**0.002**	**1.083**	**0.031**
Spatial lag^a^					**0.029**	**0.000**	**0.019**	**0.000**

Numbers highlighted in bold indicate statistically significant estimates in each model

^a^indicates a significant Wald test of parameter stability over time

## Results

### Characteristics of the samples

Figure 2 shows the average annual utilization rates for the FFS Medicare cohorts defined for the two periods. Many counties show annual utilization rates of less the 8%. However, it is apparent when comparing the figures that utilization rates increased over time, increasing the geographic coverage of counties with more than 8% average annual utilization.

Table 1 summarizes the set of supply and demand conditions used in the regression modeling. Sample statistics show means and standard deviations for the county-level data, as well as data sources. The average annual endoscopy utilization among the traditional Medicare enrollees increased slightly over time, from 7.4 to 7.9 percent. The average percentage across counties of all Medicare beneficiaries in the youngest and the oldest age groups increased, and the percentage of vulnerable people eligible for extra benefits (DUAL, disabled, ESRD) increased from 20.9 to 27.9 percent. The percentage of over age 65 with graduate or professional degrees increased slightly, as did population density, and the percent of the Medicare Part A coverage market increased from 14.1 to 14.8. All health market conditions, including MA plan penetration were fairly stable.

### Estimates from multivariate analyses

Table 2 presents the results from both ordinary least squares (OLS) and SSUR regressions. The SSUR models include a spatial lag parameter in addition to other coefficient estimates. The results are very similar across the model types, where statistically significant results for each model are highlighted in bold font (Table 2). For both models, the Lagrange Multiplier test (not shown) allowed us to conclude that the SSUR model improved efficiency significantly as compared to an unpooled model, and parameter-specific Wald tests found many significant changes in parameters over time, as indicated by superscripted lowercase letter a. The significance of changes over time were consistent across the OLS and the spatial lag regressions. The spatial lag estimate is fairly small but statistically significant; the small lag effect is consistent with the similarity of estimates across the OLS and SSUR models.

First we discuss the demographic characteristics of the Medicare population, and how these are associated with the average annual endoscopy utilization rates in the counties they represent. Places with higher percentages of older Medicare populations saw lower screening rates, and they dropped significantly over time. Places with higher percentages of Medicare-eligible females had significantly higher rates in the later period, climbing from no difference in rates in the early period. Places with higher percentages of African Americans had higher rates in both periods. Places with higher percentages of Hispanics had lower rates in the early period, but no difference in the later period. Places with higher percentages of ‘all other’ populations (these are dominated by American Indian enclaves) exhibited significantly lower rates and these declined even more over time. Places with a higher percentage of vulnerable eligibles had significantly lower rates, and this disparity increased significantly over time.

Next we discuss market contextual factors. Places with higher percentages of elderly with graduate or professional degrees had higher utilization rates, but this disparity decreased significantly over time. Places with higher importance of Medicare in the market exhibited higher rates, and this disparity increased significantly over time. Places with higher average poverty exhibited lower rates, but this disparity did not change over time. Places with greater distance to endoscopists exhibited lower rates, and this remained steady over time. Places with a more competitive endoscopy environment exhibited significantly higher rates, and this was stable over time. Places with higher MA plan penetration saw significantly lower screening rates in the early period, but this disparity became statistically weaker by the later period and the change over time was not statistically significant.

## Discussion

This study demonstrated there was an increase in endoscopy utilization among FFS Medicare after implementation of the MMA. However, the increase was not uniform across geography and various contextual and compositional market factors predicted the observed changes in utilization noted over time. Disparities (lower utilization rates) for places with higher percentages of Medicare eligible women and Hispanics decreased over time. Disparities (lower utilization rates) for places with higher percentages of ‘other’ races or ethnicities – dominated by American Indian enclaves - increased over time. Places with the highest percentages of older age groups saw significant declines over time in their screening rates, which is appropriate as screening guidelines suggest cessation of screening after age 75 because risks may outweigh benefits. Places with higher percentages of vulnerable beneficiaries exhibited lower screening rates that became even lower over time, which suggests a widening disparity over time for this more vulnerable group who may have greater difficulty undergoing the procedure and/or greater risk of complications from the procedure. Overall, the changing composition of the Medicare eligible population helped predict the net effects of supply and demand interaction on area utilization rates.

The spatial lag parameter was statistically significant and positive, suggesting that endoscopists establishing services in one county were aware of competitors in nearby counties. However, this effect diminished significantly over time, reflecting the fact that utilization tended to be geographically concentrated in the early period and became less concentrated over time as the endoscopy services diffused to underserved and new market areas (Fig. 2).

Turning to contextual market factors, Managed care spillovers were significant and negative in the early period, but became weaker and statistically insignificant over time. The significantly negative spillover effect in the earlier period is consistent with findings from an earlier paper that looked at colonoscopy and sigmoidoscopy separately. That paper found that managed care spillovers were significantly negative for colonoscopy, but significantly positive for sigmoidoscopy - the older, simpler, less risky and less costly procedure [5]. During the early period, following expansion of Medicare coverage for colonoscopy in 2001, the newer colonoscopy service diffused and came to dominate FFS Medicare endoscopy markets by 2005. The national guidelines had not yet been established during the early period, although cost-effectiveness evidence was mounting in favor of a combined use of the two endoscopy procedures, culminating in a complex screening protocol established as the gold standard in 2008 [11]. Findings here suggest that the managed care spillover seemed to have restraining influences on colonoscopy utilization at first, but this gradually diminished as the procedure gained medical acceptance over the period, and by the later period, exhibited no significant spillover effects.

During the time of this study (2001-2009), a substantial out-of-pocket copayment was required for endoscopic CRC screening. With the implementation of the MMA in 2006, subsidized coverage for prescription drugs would perhaps relax budget constraints for seniors, making such copayments more affordable, and increase utilization rates. Also, with drug coverage available in all MA plans, enrollments escalated and MA plans moved into previously underserved Medicare markets (Fig. 1). With this expansion, dissemination of best practices regarding endoscopic CRC screening might have spread into these newer, less urban markets. Such dissemination could be accompanied by spillover effects encouraging the use of endoscopy for CRC screening by FFS Medicare enrollees in those markets. With both effects in play, we expected to see an increase in utilization rates by FFS Medicare insureds over time, and an increasing MA spillover onto the FFS Medicare beneficiaries over time.

## Conclusions

The data show that annual utilization rates of endoscopy among FFS Medicare enrollees did increase over time, and findings suggest that managed care spillover effects did increase over time (from retraining to non-restraining). This is great news, because as shown in Fig. 2, overall utilization rates are low, and much improvement is needed in many areas of the country to encourage these recommended gold-standard CRC screening services. These findings suggest that policies such as those enacted in the Affordable Care Act of 2010 - which prohibited copayments for CRC screening by endoscopy for enrollees in private insurance or Medicare - are expected to result in higher rates of screening uptake, which would be an important topic for a future study.

## Abbreviations

CMS: Centers for medicare and Medicaid Services
CRC: colorectal cancer
ESRD: end stage renal disease
FFS: fee for service
MA: Medicare Advantage
MMA: Medicare Modernization Act
OLS: ordinary least squares
SSUR: spatial seemingly unrelated regressions
USPSTF: United Stated Preventive Services Task Force
